# Changes in the Text

**DOI:** 10.1001/jamahealthforum.2026.0664

**Published:** 2026-03-27

In the Viewpoint titled “Law, Politics, and Expert Panels at the US Food and Drug Administration,”^1^ published on February 13, 2026, citations were added throughout the text to support direct quotes. This article has been corrected.
